# *Annona muricata* Leaf Extract Triggered Intrinsic Apoptotic Pathway to Attenuate Cancerous Features of Triple Negative Breast Cancer MDA-MB-231 Cells

**DOI:** 10.1155/2018/7972916

**Published:** 2018-07-17

**Authors:** Jee Young Kim, Thien T. P. Dao, Kwangho Song, Sait Byul Park, Hyeri Jang, Min Kyoung Park, Soo Ueng Gan, Yeong Shik Kim

**Affiliations:** ^1^College of Pharmacy and Natural Product Research Institute, Seoul National University, Seoul 08826, Republic of Korea; ^2^Civil Appellate Division, Seoul Central District Court, Seoul 06594, Republic of Korea; ^3^Westmoreland Alternative Medicine Association, 1801 Wilshire Boulevard, Los Angeles, CA 90057, USA

## Abstract

*Annona muricata *L., known as graviola, is an evergreen plant of the tropical regions and is a rich source of natural products. Graviola has various biological activities, and it is best known for its anticancer activity. This study aimed to investigate the effects of crude graviola extract* in vitro* on breast cancer cells; in particular, we aimed to identify an agent against triple negative breast cancer (TNBC). We used the TNBC MDA-MB-231 cell line as the experimental model and the ER(+) non-TNBC MCF-7 breast cancer cell line as the control. We identified annonaceous acetogenins, including annonacin isomers, characteristic to this plant by using liquid chromatography tandem mass spectrometry (LC/MS/MS). We observed a significant decrease in the cell viability in both cell lines within 48 h, whereas impaired cell motility and invasiveness were observed only in the MDA-MB-231 cell line. While the MCF-7 cells showed an ER-dependent mechanism of apoptosis, the apoptosis of MDA-MB-231 cells was governed by an intrinsic apoptotic pathway triggered by graviola leaf extract (GLE).

## 1. Introduction

Breast cancer is the second most common cancer in the world and by far the most frequent cancer among women, with an estimated 1.67 million new cases diagnosed in 2012 (accounting for 25% of all cancers). Breast cancer is the most common cancer among women both in more and less developed regions, and slightly higher number of cases have been reported in the less developed regions (approximately 883,000 cases) than in the more developed regions (approximately 794,000). Breast cancer ranks fifth among the causes of death from cancer and is the most frequent cause of cancer-related death in women in less developed regions (approximately 324,000 death; 14.3% of total) and it ranks second after lung cancer as a cause of cancer-related death in more developed regions (approximately 198,000 death; 15.4%) [1].

Breast cancer is not a single disease; there are a minimum of four molecular subtypes and 21 histological subtypes of breast cancer with all variable risk factors. Breast cancer is classified into different molecular subtypes using routine biological markers, i.e., estrogen receptor (ER), progesterone receptor (PR), and human epidermal growth factor receptor 2 (HER2). Approximately 12% of patients with breast cancer are ER(−), PR(−), and HER2(−) [2]; this subtype is known as the triple negative breast cancer (TNBC). Patients with TNBC have poorer prognosis and higher risk of recurrence than patients with ER(+) or HER2/neu-(+) breast cancer; TNBC is a clinically aggressive disease associated with distant recurrence and high rates of visceral and central nervous metastases [3, 4]. The standard treatment for TNBC includes chemotherapy with microtubule stabilizers and platinum agents alone or in combination with other therapeutic options such as surgery and radiotherapy. Patients with TNBC do not benefit from hormone receptor-targeted therapies such as tamoxifen and trastuzumab, which are used for ER(+) and HER2(+) breast cancer; therefore, inhibitors of common apoptotic pathways, e.g., poly (ADP-ribose) polymerase (PARP) and mammalian target of rapamycin (mTOR), and inhibitors of cancer cell metabolism, e.g., antiangiogenesis agents, have been used for the treatment of TNBC [5]. A recent study has shown positive effects of immunotherapy for TNBC; however, further studies are required to establish the efficacy of immunotherapy for TNBC [6].

The limitations of the existing therapeutic options for TNBC warrant the discovery of novel molecular agents for treating TNBC. Graviola (*Annona muricata *L.), also known as soursop, guanabana, or Brazilian pawpaw, is an evergreen plant of tropical and subtropical regions and is a rich source of natural products [7]. The phytochemicals derived from graviola have various biological activities, including anticancer, antiarthritic, and antiparasitic activities [7]. Annonaceous acetogenins (AAs) are the metabolites isolated from the plants belonging to the genus* Annona*; the AAs characterized by a terminal gamma-lactone ring and a single tetrahydrofuran (THF) ring in the middle of aliphatic chains are the active ingredients responsible for the cytotoxic effects of graviola. The AAs isolated from plants belonging to the family Annonaceae exert their effects through the inhibition of mitochondrial complex I, namely, NADH: ubiquinone oxidoreductase [8]. Currently, more than 100 AAs have been identified and isolated; pure AAs have been screened for their anticancer activity, and the studies performed in the previous three decades have shown promising results.

Recent* in vitro* studies indicate that the crude extract of* A. muricata* alone can be used as an alternative chemotherapy against pancreatic cancer [9], prostate cancer [10], and breast cancer [11]. Here, we examined the effects of* A. muricata* leaf extract on TNBC* in vitro *using the MDA-MB-231 cell line and compared the results with those obtained using the ER(+) breast cancer cell line MCF-7 (control) to determine the mechanism underlying apoptosis. Our results may provide insights into a targeted therapy for TNBC and establish the therapeutic potential of this plant beyond its status as a functional food.

## 2. Methods and Materials

### 2.1. Preparation of Graviola Leaf Extract (GLE)

GLE (*aq*) made of* Annona muricata *leaves (Philippines) was provided from Westmoreland Alternative Medicine Association. Professor Youngbae Suh in our department organoleptically identified the specimen of* A. muricata*. The voucher specimen (2017-25) has been deposited in the herbarium of Natural Products Research Institute, SNU. To prepare aqueous concentrate, graviola leaves were air-dried and finely milled into powder. Collected graviola leaf powder (1 kg) was blended with purified water (5 L) and then processed in a vacuum extractor (COSMOS-660, Kyung Seo Co., Korea) under proper heating cycle. Produced aqueous suspension was further concentrated (over 40°Brix) so approximately 100 mL of the final GLE (*aq*) could be acquired from 1 kg of dried* Annona muricata* leaves. Concentrated GLE (*aq*) was filtered and counter-extracted in methylene chloride (1:1 v/v) and organic solvents were evaporated with N_2_ gas. Remaining products were suspended then further diluted in DMSO (100, 50, and 25 mg/mL) for* in vitro* treatment [9] or suspended in methanol (3000 *μ*g/mL) for LC/MS/MS analysis.

### 2.2. LC/MS/MS Analysis of GLE

Active ingredients of* Annona muricata* leaf extract were analyzed using the HPLC system (Agilent Technologies, USA) linked to a 6530 ESI-Q-TOF MS spectrometer (Agilent Technologies). The reconstituted sample was prepared as 3000 ppm in MeOH (Section 2.1); we injected 5 *μ*L of the reconstituted sample into the LC/MS/MS system. LC separation was performed on an INNO C18 column (2.0 × 50 mm, 3.0 *μ*m) with mobile phase A (0.1% formic acid in water) and mobile phase B (0.1% formic acid in acetonitrile). The gradient program (A/B: 9:1 →1:9 in 25 min) was followed by 5 min 100% B washing, and the column was then equilibrated with 10% B for 5 min while the flow rate was maintained at 0.30 mL/min. Subsequently, we obtained the mass spectra under positive electrospray ionization (ESI) with an ion spray voltage of 4000 V. The source temperature was 350°C. The flow speed of gas was 10 L/min while the pressure of nebulizer was 30 psi. Full-scan mass spectra were acquired within an* m*/*z* range of 50−1400 in the MS mode. The data obtained were analyzed using MassHunter Qualitative Analysis software (Agilent Technologies).

### 2.3. Cell Culture

TNBC cell line MDA-MB-231 and ER(+) breast cancer cell line MCF-7 were acquired from American Type Culture Collection (ATCC). Breast cancer cells were cultured in DMEM (CM002-250, GenDEPOT) medium supplemented with a 25-mM HEPES buffer (CA011-010, GenDEPOT), 10% (v/v) fetal bovine serum (FBS), and 1% (v/v) PenStrep (the final concentration of antibiotics to be 100 U/mL penicillin and 100 *μ*g/mL streptomycin). Cells were maintained at 37°C humidified atmosphere with 5% CO_2_.

### 2.4. Cell Viability Assay

Cytotoxicity of GLE was measured by MTT assay. MDA-MB-231 (5.0 × 10^4^ cells/well) and MCF-7 (5.0 × 10^4^ cells/well) cells were seeded with increasing concentration (0, 50, 100, 200 *μ*g/mL) of GLE and cultured on a 24-well plates. After 48 hours, MTT reagent (M2128, Sigma) stock solution (5 mg/mL in DPBS) was added to each well (final concentration of MTT 0.5 mg/ml) and cells were incubated for additional 4 hours. Medium was removed then replaced with DMSO to dissolve MTT formazan. The cell viability of each experimental group was measured at 540 nm absorbance filter on a microplate reader and results were obtained from three independent experiments.

### 2.5. Clonogenicity Assay

MDA-MB-231 cells were seeded on a 24-well plates (1000-2000 cells/well) and then exposed to different concentrations of GLE (0, 50, 100, and 200 *μ*g/mL). After 24-hour or and 48-hour incubation, cells were carefully rinsed and cultured in the fresh medium. Surviving adherent cells were further cultured for additional 10 days to gain visible colonies. Colonies were rinsed with DPBS, fixed with 3.7% formalin, stained with 0.5% crystal violet dissolved in 20% MeOH [12], and photographed.

### 2.6. Wound-Healing Assay

MDA-MB-231 and MCF-7 cells were seeded on 24-well plates to reach 80–90% confluency after overnight incubation. Once the cells formed monolayer, cells were scratched with sterilized pipette tips. Detached cells were removed by careful washing and medium was changed exposing scratched cell layer to different concentrations of GLE (0, 50, 100, and 200 *μ*g/mL). After 24-hour incubation, recovered monolayers were photographed under a CKX41 microscope (Olympus, Japan) at a 40× magnification. Images were processed with ProgRes CapturePro software v.2.8.8 (JENOPTIK Optical Systems, USA).

### 2.7. Cell Invasion Assay

MDA-MB-231 (5.0 × 10^5^ cells/well) cells were seeded on transwell inserts with 8.0-*μ*m pore size (SPL Life Sciences Co., Seoul, Korea) coated with properly diluted Matrigel with serum-free DMEM (1:10) prior to use. Cells were exposed to different concentrations of GLE (0, 50, 100, and 200 *μ*g/mL) at a starved condition (FBS-free DMEM). After 24-hour incubation, both sides of the transwell inserts were washed with DPBS and cells were fixed with 3.7% formalin, permeabilized with MeOH, and then finally stained with 2% crystal violet solution (*aq*). Stained cells were observed and photographed under a CKX41 microscope at a 100× magnification.

### 2.8. Cell Cycle Analysis

Cell cycle phases were assessed as described elsewhere [14]. In brief, MDA-MB-231 (2.0 × 10^6^ cells/well) and MCF-7 (2.0 × 10^6^ cells/well) cells were seeded in six-well plates then exposed to GLE (0, 50, 100, and 200 *μ*g/mL) for 24 hours. Incubated cells were collected, washed with DPBS, fixed with cold 70% ethanol, and then stored at −20°C for a minimum of 24 hours. Ethanol was removed by centrifugation right before the analysis and cell pellets were repetitively washed with DPBS. Cellular RNA was removed by incubation with RNase (200 *μ*g/mL) at 37°C for 30 min, then cellular genomic DNA was stained with propidium iodide (PI, 50 *μ*g/mL) for another 30 min in RT. Cells were sorted on FACSCalibur flow cytometry (BD Biosciences) according to detected signals in FL2 channel (ext. 488 nm, emi. 564–606 nm) while data was analyzed with Cell Quest Pro software.

### 2.9. Western Blotting

Pellets of MCF-7 and MDA-MB-231 cells were collected through centrifugation, briefly washed with DPBS, and then homogenized with a lysis buffer (20 mM HEPES, pH 7.6, 350 mM NaCl, 20% glycerol, 1% NP-40, 1 mM MgCl_2_, 0.5 mM EDTA, 0.1 mM EGTA, 1 mM DTT, 1 mM PMSF, and a protease inhibitor cocktail). After vigorous vortexing and ultracentrifugation, we denatured protein lysates in the supernatants through the process of boiling with 5X loading dye. Proteins were separated via SDS electrophoresis with 10% gel, followed by a transfer of separated protein bands on the PVDF membrane. Transferred protein bands were blocked with 5% BSA then incubated at 4°C overnight with the following primary antibodies and given dilution rates; *β*-actin (C4) HRP (mouse monoclonal, Santa Cruz 47778) as 1:2000, PARP (rabbit, GeneTex 100573) as 1:3000, cytochrome C (rabbit, Epitomics 1896-1) as 1:500, ER alpha (rabbit, Epitomics 1115-S) as 1:1000, and HER2 (rabbit, Epitomics 2521-1) 1:1000 as optional. After the culture with primary antibodies, the protein bands were labeled with a goat anti-rabbit IgG (H+L) HRP secondary antibody 1:3000 for one hour at room temperature, with the exception of *β*-actin. The protein bands were visualized via the EZ-Western (DG-W500, Daeillab Service) ECL solution, then photographed on the LAS-1000 imaging system (Fujifilm, Japan), and operated by Image Reader LAS-1000 Lite V1.5, while processing images with MultiGuage V3.0 software.

### 2.10. Confocal Microscopy

MDA-MB-231 (5.0 × 10^4^ cells/well) and MCF-7 (5.0 × 10^4^ cells/well) cells were seeded on confocal dishes (SPL Life Sciences Co., Seoul, Korea) and cultured with different concentrations of GLE (0, 50, 100, and 200 *μ*g/mL) in the incubator. After 24 hours, medium was replaced with a live staining cocktail of 10 *μ*M 2′7′-dichlorofluorescein diacetate (denoted as DCF-DA, ≥ 95%, Sigma), 50 *μ*g/ml propidium iodide (≥ 94.0%, Sigma), and 1 *μ*g/mL bisBenzimide H 33342 trihydrochloride (≥ 97.0%, Sigma). After 20 minute incubation with fluorophores, cells were washed twice and stained cells were observed on a TCP SP8 confocal microscope (Leica, Germany).

### 2.11. Statistical Analysis

Student's* t*-test was used to determine statistical significance between the control (0 *μ*g/mL, DMSO) and experimental (50, 100, and 200 *μ*g/mL) groups in MTT assay. Statistical analysis was performed using Microsoft Office Excel 2007 under the condition of equal sample size and unequal variances. Calculated* p*-values < 0.05 were considered statistically significant.

## 3. Results

### 3.1. GLE Contains Characteristic AA Isomers of* Annona muricata* Leaves

**Figure 1:**
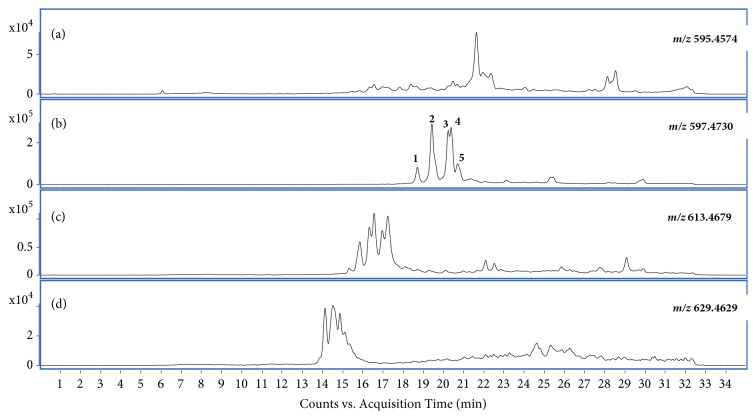
*The extracted-ion chromatograms obtained for the A. muricata leaf extract using HPLC-QTOF*. (a) EIC of m/z 595.4574 (± 10.0 ppm) [C_35_H_62_O_7_+H]+; (b) EIC of m/z 597.4730 (± 10.0 ppm) [C_35_H_64_O_7_ +H]+; (c) EIC of m/z 613.4679 (± 10.0 ppm) [C_35_H_64_O_8_ +H]+; (d) EIC of m/z 629.4629 (± 10.0 ppm) [C_35_H_64_O_9_ +H]+.

Previous studies have shown that AAs are abundantly present in the leaf, stem, and root of* A. muricata*. The results of matrix assisted laser desorption/ionization time of flight mass spectrometry (MALDI-TOF MS) of products derived from different parts of this plant showed the presence of four representative AAs in the leaves of graviola, which may have the common skeleton characterized by a long C32 alkyl backbone ending in a *γ*-lactone: C_35_H_62_O_7_; C_35_H_64_O_7_, C_35_H_64_O_8_, and C_35_H_64_O_9_ [15].  Figure 1 displays the obtained extracted-ion chromatograms (EIC) showing the series of peaks with* m/z* values corresponding to the four selected molecular formulas. The most abundant peaks can be found in the EIC of C_35_H_64_O_7_ (*m/z* 597.4695 ± 10.0 ppm) which corresponds to many monotetrahydrofuran (THF) acetogenins found in* A. muricata,* including the annonacin-type AAs [16].

**Figure 2:**
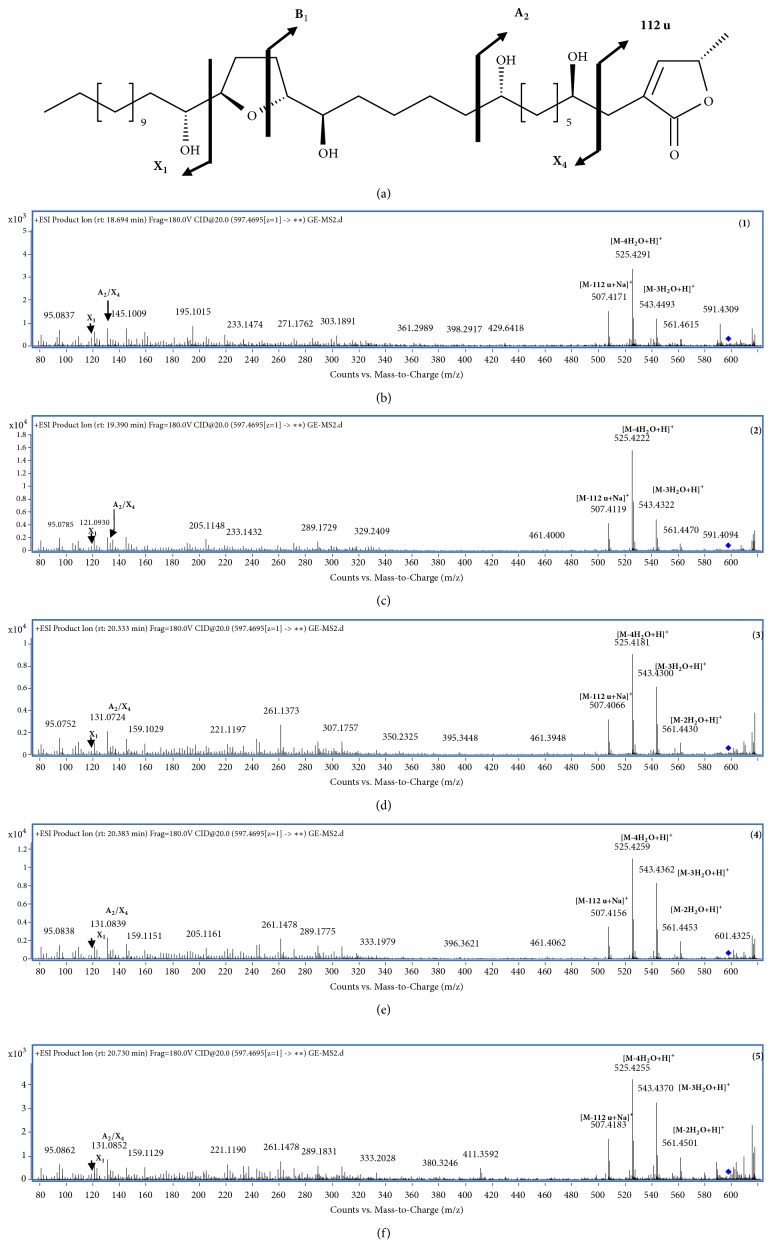
*Product ion chromatograms of the formula C*_35_ *H*_64_ *O*_7_. (a) Structure of annonacin (596.4652 u) and the nomenclature of its fragments [13], in which the A2/X4 and B1/X4 fragments are named for the patterns between X4 and A2 or B1, respectively. (b)–(f) Product ion chromatogram of [C_35_H_64_O_7_ +H]^+^ at five main peaks displayed the fragment ions related to annonacin-type compounds.

According to Allegrand* et al.* (2010), the characteristic ions are adequate to identify and localize the functional groups on the alkyl chain, including the presence of the 112 u loss (the lactone ring fragment), H_2_O losses, and the typical fragment series [17]. Since AAs have homogeneous structures, the results of MS fragmentation allowed us to confirm the presence of some AAs in the GLE through structural ion peaks that representative for the loss of terminal *γ*-lactone ring [M-112u+Na]^+^ and the number of the hydroxyl groups [M-xH_2_O+H]^+^ in the structures (Figure 2 and Figure S1-S3 in Supplementary Materials). Notably, the five main peaks corresponding to C_35_H_64_O_7_ (*m/z* 597.4695) shared characteristic fragments that were similar to the previously described patterns for the annonacin structure (Figure 2(a)). In particular, the remarkable X4 fragment (*m/z* 507.4006, [M-112u+Na]^+^) properly characterizes the loss of the terminal lactone ring; the B1/X4 fragment (*m/z* 243.1384) might indicate that the THF is located between C16 and C19 flanked by two hydroxyl groups at C15 and C20, whereas the X1 ions (*m/z* 199.1475) suggest the presence of a hydroxyl group at C10 on the alkyl chain consistent with the results reported in a previous study [13, 18] (Figures 2(b)–2(f)). Thus, the identification of AAs in the crude extract used in this study was achieved to some degree while precise calculation of the molecular content remains to be performed.

### 3.2. GLE Suppresses Proliferation and Clonogenicity of MDA-MB-231 Cells

**Figure 3:**
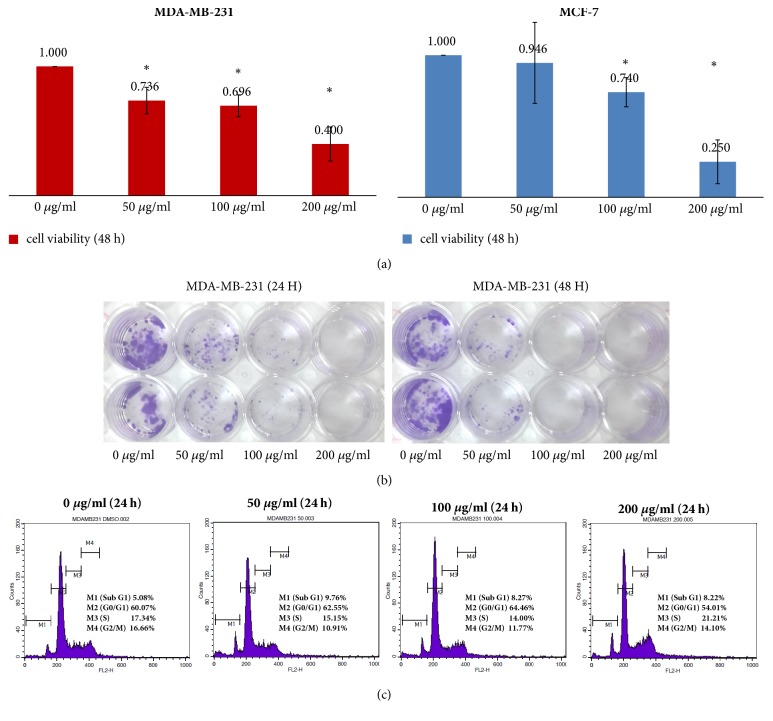
*Effects of GLE on cell viability, clonogenicity, and cell cycle*. Breast cancer cells were treated with GLE at various concentrations (0, 50, 100, and 200 *μ*g/mL) for 48 hours and their cell viability was measured by MTT assay (a). MDA-MB-231 cells treated with GLE were allowed to grow into visible colony for additional 10 days (b). MDA-MB-231 cells treated with GLE were fixed and sorted by genomic DNA contents (c).

At the beginning of the biological assays, cell viability was tested on breast cancer cells to verify cytotoxicity of GLE and GLE led to decreased viability of MDA-MB-231 (Figure 3(a), left) and MCF-7 (Figure 3(a), right) cells. According to a previous study, 48-hour treatment of graviola extracts induced significantly decreased cell viability in FG/COLO357 and CD18/HPAF pancreatic cancer cells [9]. Surprisingly, results from the MTT assay indicate a significant decrease in breast cancer cells' viability after 48-hour incubation with increasing concentrations (0, 50, 100, and 200 *μ*g/mL) of GLE (Figure 3(a)). Overall, GLE showed cytotoxicity on breast cancer cells in a dose-dependent manner and suppressed cell proliferation.

A more vigorous colony formation was expected in TNBC cell line MDA-MB-231 than in non-TNBC cell line MCF-7. As expected, visible colonies of MDA-MB-231 could be identified within 10 days, unlike MCF-7 (data not shown), and MDA-MB-231's clonogenicity was shrunk by GLE treatment as observed (Figure 3(b)). It is noteworthy that the number of MDA-MB-231 colonies substantially decreased with increasing concentration of GLE. Total amelioration of the clonogenicity could be observed within 24 hours to the 100 *μ*g/mL dose and 48 hours to the 50 *μ*g/mL (Figure 3(b)). In addition, cell cycle analysis after 24-hour treatment with GLE demonstrated the change of cell population in different phases. While there was no significant change in the populations of other cell cycle phases (S and G2/M), graviola treatment resulted in the accumulation of sub-G1 population which represents dead cells (Figure 3(c)). Similar to the MTT assay data (Figure 3(a), right), the results of cell cycle analysis further support GLE's cytotoxic effects on MDA-MB-231 cells.

### 3.3. GLE Decreases Motility and Invasiveness of MDA-MB-231 Cells

**Figure 4:**
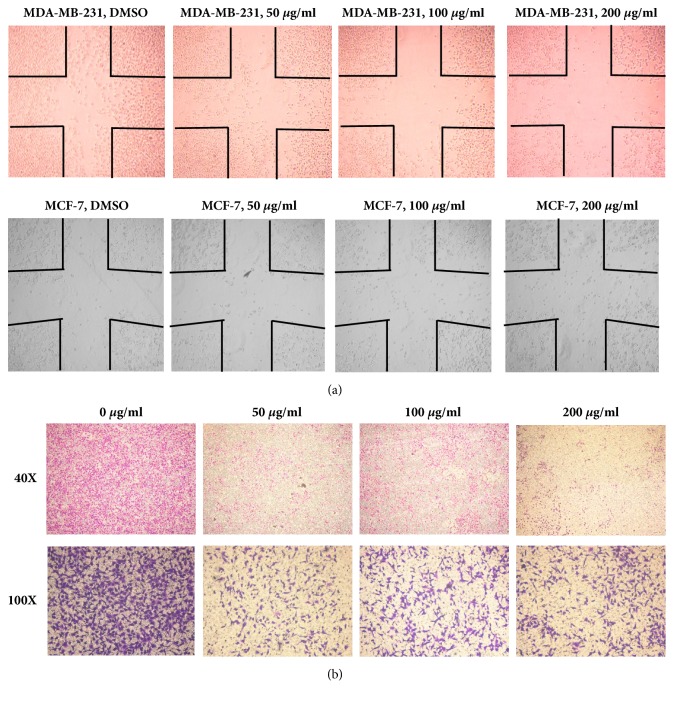
*Effects of GLE on cell motility and invasion of breast cancer cells*. Recovery of scratched cell monolayer in MDA-MB-231 (upper) and MCF-7 (lower) (a) and transwell invasion of MDA-MB-231 cells (b) were observed after 24-hour exposure with various concentrations of GLE.

Possible effects of GLE on motility of breast cancer cells were further analyzed. As seen in the results from the wound-healing assay, the uncovered area increased in treated MDA-MB-231 cells with the increase of GLE concentrations (0, 50, 100, and 200 *μ*g/mL) (Figure 4(a), upper row). Cellular motility was seldom affected in MCF-7 cells upon treatment (Figure 4(a), lower row), but cellular morphology was obviously altered in both cell lines (Figure 4(a)).

Subsequently, the effects of GLE on cellular invasiveness of MDA-MB-231 cells were further investigated. Invasion of MDA-MB-231 cells over the Matrigel matrix of transwell chamber was significantly suppressed upon GLE treatment (0, 50, 100, and 200 *μ*g/mL) though not strictly in a dose-dependent manner (Figure 4(b)). Taken together, 24 h exposure of GLE resulted obviously decreased motility and invasiveness in MDA-MB-231 TNBC cells.

### 3.4. GLE Triggers Intrinsic Apoptotic Pathway through ROS Formation in MDA-MB-231 Cells

**Figure 5:**
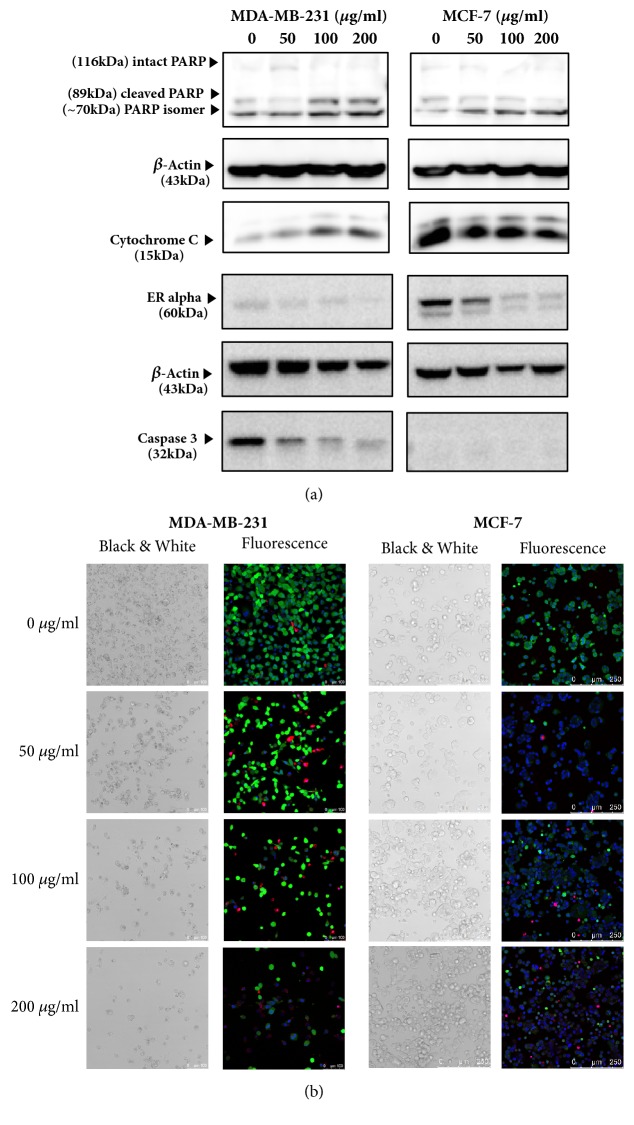
*Different apoptotic mechanisms in breast cancer cell lines caused by GLE*. Effects of GLE on apoptosis of MDA-MB-231 (left) and MCF-7 (right) cells were analyzed by WB (a) and cellular ROS formation (green fluorescence, DCFDA) in MDA-MB-231 (left) and MCF-7 (right) were visualized by CLSM. Scale bar denotes 100 *μ*m (left, MDA-MB-231) and 250 *μ*m (right, MCF-7), respectively (b).

The mechanisms of cell death induced by GLE were investigated by observing protein regulators of apoptosis and visualizing cellular ROS formation. According to a previous study, annonacin induced ER*α*-related apoptotic phenomena on MCF-7 cells [19]. When analyzed by Western blot, a similar pattern of apoptosis (i.e., ER*α*-related pathway) was observed in MCF-7 cells treated with crude GLE (Figure 5(a), right), possibly due to the rich presence of characteristic AAs in our GLE. Interestingly enough, the apoptotic mechanism in MDA-MB-231 cells differed from that of MCF-7 cells. MDA-MB-231 cells exposed to GLE underwent intrinsic apoptotic pathway (Figure 5(a), left) characterized by altered mitochondrial membrane potential (cytochrome C), activated caspase cascade (caspase 3), and damaged DNA (PARP). Since estrogen receptors (ERs) are absent in TNBC cell line like MDA-MB-231, a different apoptotic signaling from that of MCF-7 could well be assumed.

All of the above described apoptotic reactions induced by GLE may involve ROS formation in breast cancer cells; thus signals of ROS were detected by a confocal microscopy (Figure 5(b)). Seen from the proportion of DCFDA-positive cells (denoted as green fluorescence), cellular ROS production increased along with PI-positive apoptotic population (denoted as red fluorescence) in MDA-MB-231 cells, although treatment with a lethal concentration of GLE (200 *μ*g/mL) resulted in the rupture of cell membranes and a loss of cellular fluorescence signals. In contrast, the fluorescence signals were unaffected by the increasing concentrations of GLE in MCF-7, proving the irrelevance of the GLE treatment to the ROS formation. All in all, GLE increased intracellular ROS accompanied by mitochondrial apoptotic pathways in MDA-MB-231 cells only.

## 4. Discussion

The present study indicates that the active ingredients in the GLE have a marked anticancer effect on the MDA-MB-231 TNBC cells. TNBC is the most aggressive form of breast cancer, and the crude extract of* A. muricata* not only inhibits proliferation but also inhibits metastasis. We also identified different apoptotic pathways in MDA-MB-231 and MCF-7 breast cancer cells resulted from GLE treatment. To our knowledge, no study to date has simultaneously investigated the mechanism of apoptosis induced by the GLE in the MDA-MB-231 and MCF-7 cells.

Further, we determined the presence of AAs, which are the active ingredients of* A. muricata*, in our sample using LC/MS/MS analysis. A previous study has reported the mechanism underlying the apoptosis induced by the AA annonacin in a non-TNBC model MCF-7 [19]. Our data indicate that the TNBC cells do not undergo apoptosis via the same mechanism as that of non-TNBC cells, and thus, active ingredients apart from the well-known AAs present in GLE may be responsible for the apoptotic effects in TNBC cells.

Previous studies have reported the marked anticancer effect of plants belonging to the* Annona *species [20], particularly* Annona muricata*. The promising results have been obtained in recent studies on the efficacy of* A. muricata* leaves on colon cancer cell lines, namely, HCT-116 and HT29 [21], and the COLO-205 cell line [22] and in a preliminary clinical study [23]. The neurotoxicity caused by the consumption of the family Annonaceae should not be overseen, which is associated with Parkinson's disease [24–26]. The side effects of the GLE must be considered before for therapeutic applications. Further, the complete molecular structure of the active ingredients and the exact molecular mechanism need to be determined.

## 5. Conclusions

Taken together, we partially identified the active ingredients present in the GLE and determined their anti-TNBC activities. The crude extract of the graviola leaves induced mitochondrial apoptosis, suppressed cell proliferation, and decreased cellular motility in MDA-MB-231 TNBC cells. Further studies are required to establish the therapeutic potential of this medicinal plant beyond its status as a functional food.

## Data Availability

Mass data are available in the Supplementary Materials.

## Abbreviations

TNBC: Triple negative breast cancer
GLE: Graviola leaf extract
ER: Estrogen receptor
AA: Annonaceous acetogenin
MTT: 3-(4,5-Dimethylthiazol-2-yl)-2,5-diphenyltetrazolium bromide
LC/MS/MS: Liquid chromatography tandem mass spectrometry
MALDI-TOF: Matrix assisted laser desorption/ionization time of flight
EIC: Extracted-ion chromatogram.

## Supplementary Materials

Supplementary Materials Figure S1: product ion chromatograms of the formula C_35_H_62_O_7_. Product ion chromatograms at different retention time, which corresponds to the characterized fragments of the lactone ring ([M-112u+Na]^+^) and the number of hydroxyl groups on the alkyl chain ([M-xH_2_O+H]^+^). Figure S2: product ion chromatograms of the formula C_35_H_64_O_8_. Product ion chromatograms at different retention time, which are consistent with the characterized fragments of the lactone ring ([M-112u+Na]^+^) and the number of hydroxyl groups on the alkyl chain ([M-xH_2_O+H]^+^). Figure S3: product ion chromatograms of the formula C_35_H_64_O_9_. Product ion chromatograms at different retention time, which corresponds to the characterized fragments of the lactone ring ([M-112u+Na]^+^) and the number of hydroxyl groups on the alkyl chain ([M-xH_2_O+H]^+^).
