# Supplementary material for: Annona muricata Leaf Extract Triggered Intrinsic Apoptotic Pathway to Attenuate Cancerous Features of Triple Negative Breast Cancer MDA-MB-231 Cells
Source: Evid Based Complement Alternat Med. 2018 Jul 17;2018:7972916. doi: 10.1155/2018/7972916 (PMC6076972; doi:10.1155/2018/7972916)
Supplement: Supplementary Materials — Figure S1: product ion chromatograms of the formula C35H62O7. Product ion chromatograms at different retention time, which corresponds to the characterized fragments of the lactone ring ([M-112u+Na]+) and the number of hydroxyl groups on the alkyl chain ([M-xH2O+H]+). Figure S2: product ion chromatograms of the formula C35H64O8. Product ion chromatograms at different retention time, which are consistent with the characterized fragments of the lactone ring ([M-112u+Na]+) and the number of hydroxyl groups on the alkyl chain ([M-xH2O+H]+). Figure S3: product ion chromatograms of the formula C35H64O9. Product ion chromatograms at different retention time, which corresponds to the characterized fragments of the lactone ring ([M-112u+Na]+) and the number of hydroxyl groups on the alkyl chain ([M-xH2O+H]+). [file 7972916.f1.docx]

## Supplementary Materials


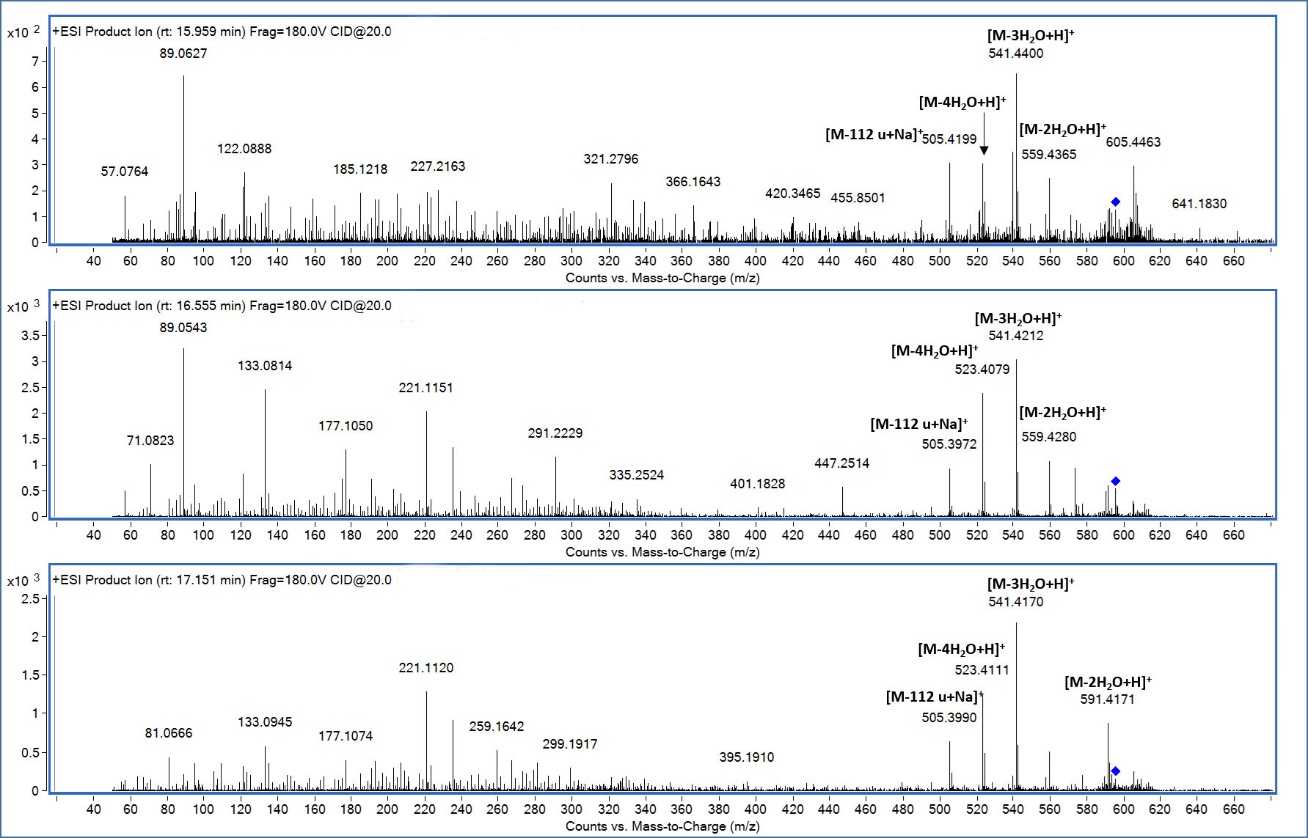


**Figure S1. Product ion chromatograms of the formula C_35_H_62_O_7_**

Product ion chromatograms at different retention time, which corresponds to the characterized fragments of the lactone ring ([M-112u+Na]^+^) and the number of hydroxyl groups on the alkyl chain ([M-xH_2_O+H]^+^).


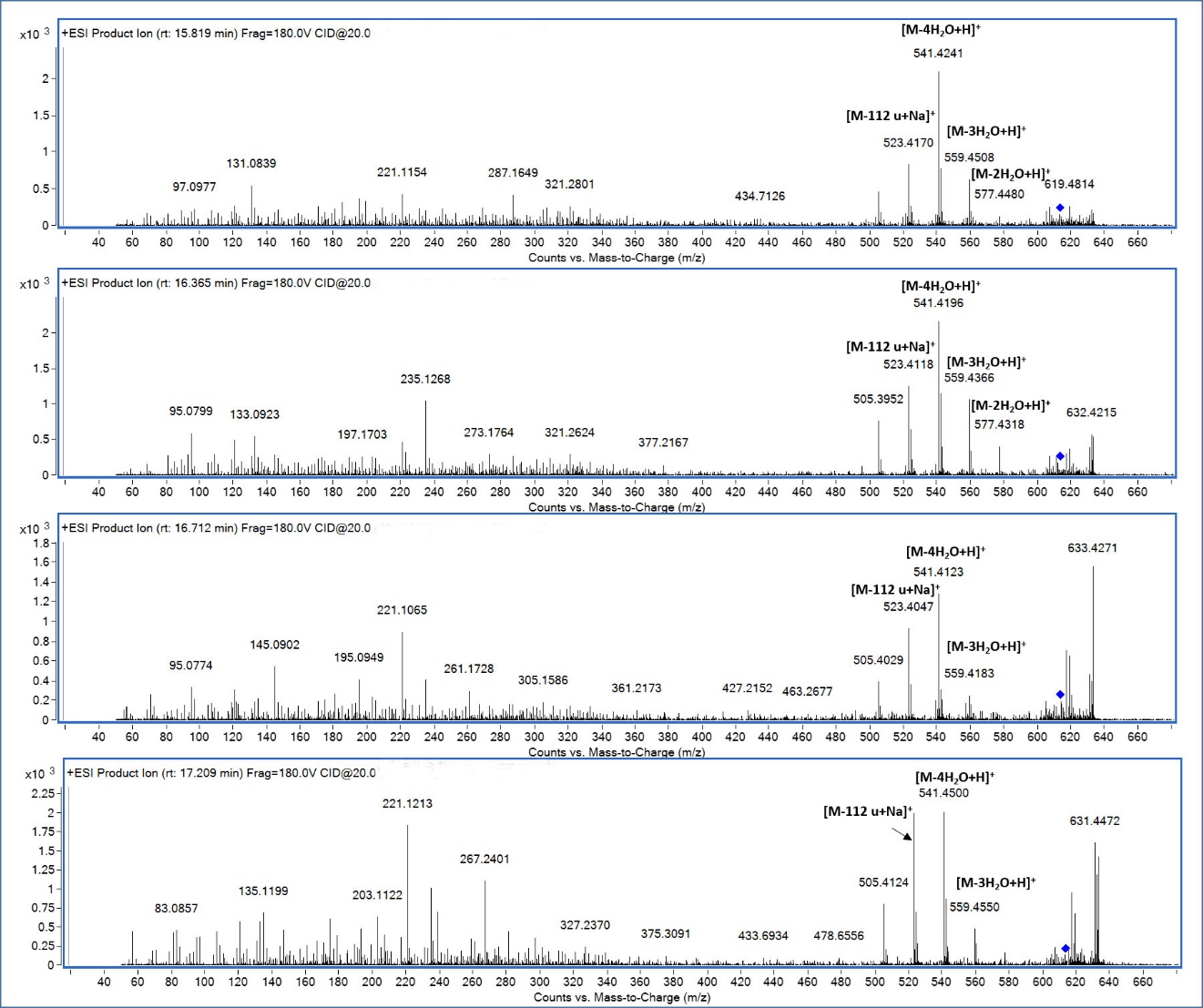


**Figure S2. Product ion chromatograms of the formula C_35_H_64_O_8_**

Product ion chromatograms at different retention time, which are consistent with the characterized fragments of the lactone ring ([M-112u+Na]^+^) and the number of hydroxyl groups on the alkyl chain ([M-xH_2_O+H]^+^).


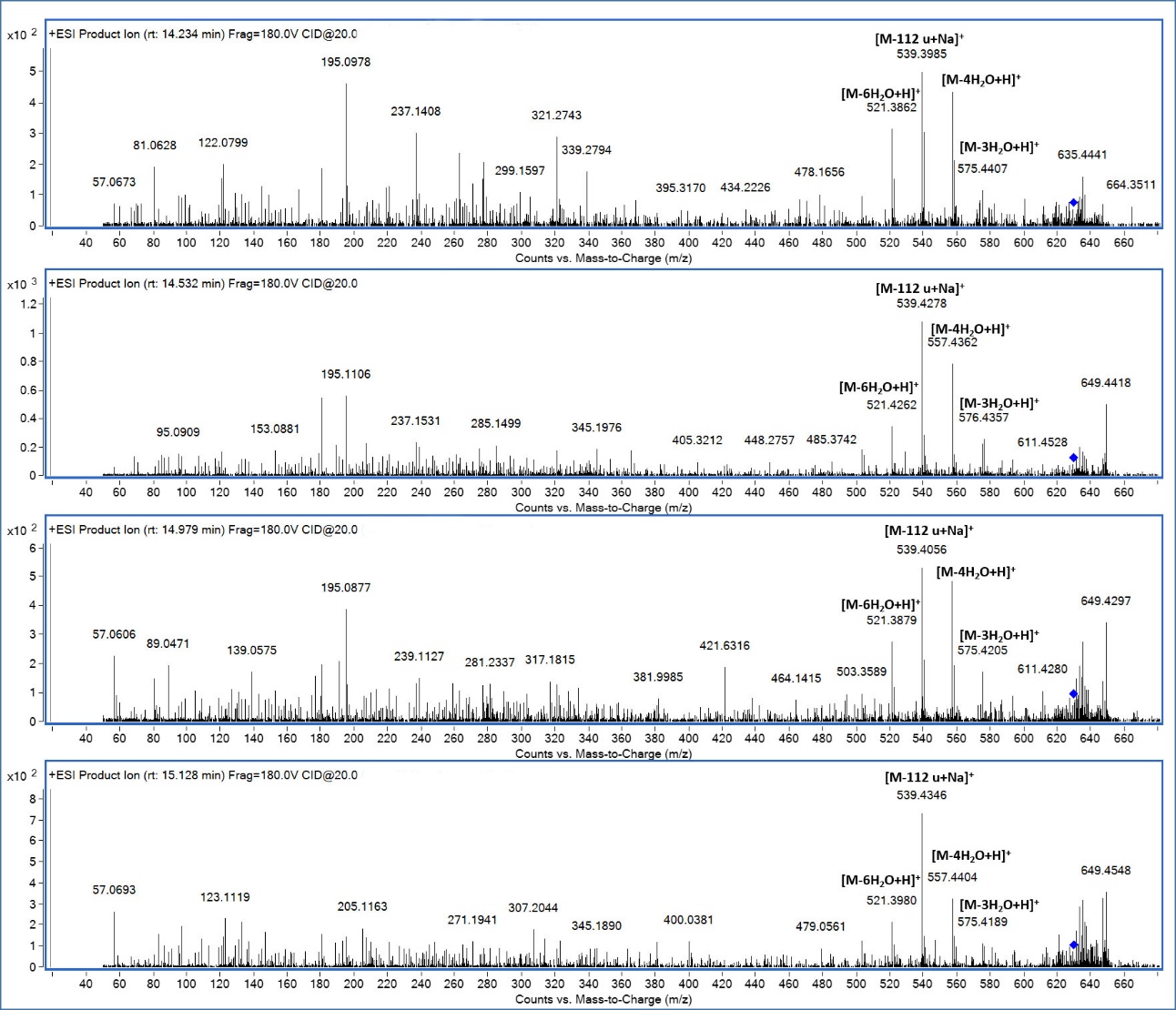


**Figure S3. Product ion chromatograms of the formula C_35_H_64_O_9_**

Product ion chromatograms at different retention time, which corresponds to the characterized fragments of the lactone ring ([M-112u+Na]^+^) and the number of hydroxyl groups on the alkyl chain ([M-xH_2_O+H]^+^).
